# The Clot Stops Here: Insights Into Venous Thromboembolism Prophylaxis Adherence From Two Cases

**DOI:** 10.7759/cureus.71866

**Published:** 2024-10-19

**Authors:** Anthony T Nguyen, Alexandra M Glaeser

**Affiliations:** 1 Internal Medicine, University of California Los Angeles, Los Angeles, USA

**Keywords:** interdisciplinary health team, pulmonary embolism (pe), quality improvement and patient safety, venous thromboembolism (vte), vte prevention

## Abstract

Pharmacologic venous thromboembolism (VTE) prophylaxis is effective and essential in the inpatient setting for medically ill patients to prevent deep venous thromboses (DVTs) and pulmonary embolisms (PEs), especially in high-risk patients such as those with limited mobility and underlying malignancy. It is less clear how the primary team, including the nurses and physicians, work together to ensure adherence to VTE prophylaxis during hospitalizations, especially amongst different institutions with varied practices. This case series describes two cases of healthcare-associated VTE after refusal of VTE prophylaxis for several days and the resulting complications, including morbidity and mortality. In light of these two cases and recognition that healthcare-associated VTE is a wider public health problem, we suggest a multi-disciplinary and standardized protocol for physicians to follow to ensure VTE prophylaxis adherence that takes into account a VTE risk assessment calculator such as the Padua or Caprini scores. Furthermore, physicians should acknowledge VTE prophylaxis refusal, counsel patients, and document their findings in high-risk patients on a daily basis. This would be a full-circle approach that encourages patient education about this commonplace yet critical practice and the potential consequences of non-adherence.

## Introduction

It is widely accepted that venous thromboembolism (VTE) prophylaxis is essential in the inpatient setting for medically ill patients to prevent deep venous thromboses (DVTs) and pulmonary embolisms (PEs), as patients’ comorbidities such as malignancy and immobility during hospitalization can portend a higher risk. VTE associated with hospitalization continues to place a high burden on hospitals. Current VTE prophylaxis guidelines suggest that assessment of risk for thromboembolism and associated risk of bleeding be individualized for each patient, and when indicated, pharmacologic prophylaxis be used in conjunction with mechanical prophylaxis [1]. However, the implementation and ownership of all the steps that it takes for a patient to receive pharmacologic prophylaxis can be more convoluted. VTE prophylaxis is often thought of as a “checklist” item on a set of inpatient admission orders and can often be overlooked by primary teams. Though the prescribing provider has a responsibility when putting in orders, it is less clear how a primary team works together to ensure adherence to pharmacologic VTE prophylaxis. This commentary discusses two cases of healthcare-associated large pulmonary emboli that led to morbidity and mortality. It examines the VTE prophylaxis process preceding these events, highlighting areas for potential improvement.

## Case presentation

Case 1

**Figure 1:**
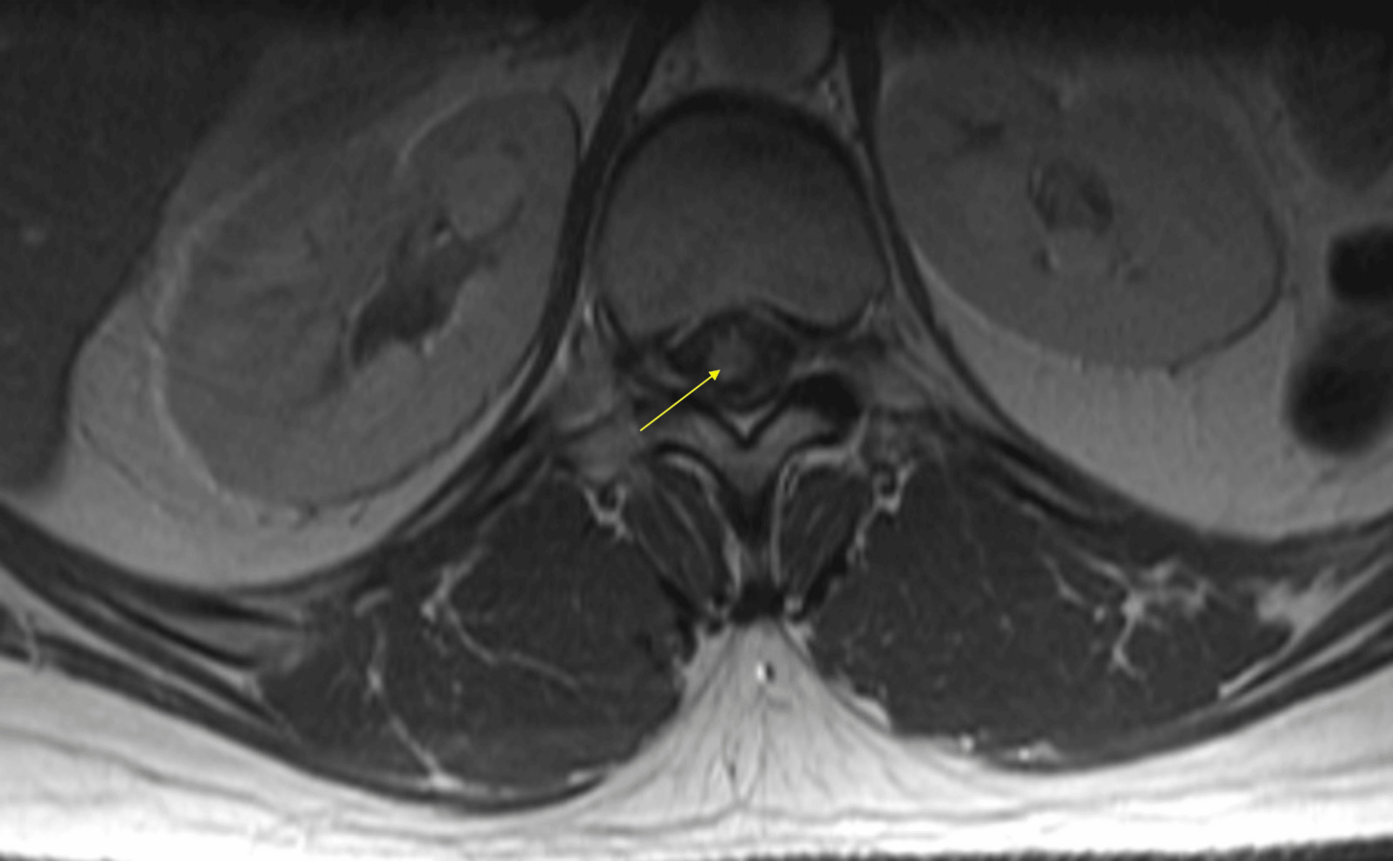
MR thoracic spine demonstrating a spinal cord nodule (yellow arrow) at the T11-T12 level consistent with metastatic atypical teratoid rhabdoid tumor with central nervous system involvement. The patient was found to have extensive leptomeningeal disease involving the cervical, thoracic, and lumbar spines.

**Figure 2:**
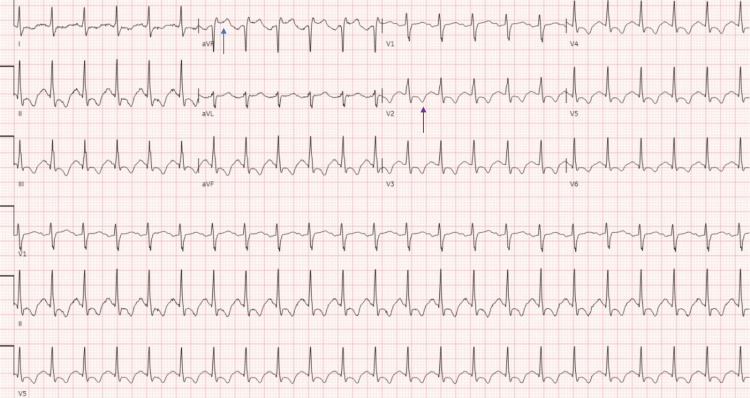
Electrocardiogram (ECG) on hospital day four showed ST elevation in aVR (blue arrow), T-wave inversions, and depressions in the lateral and precordial leads (purple arrow), concerning for global ischemia.

**Figure 3:**
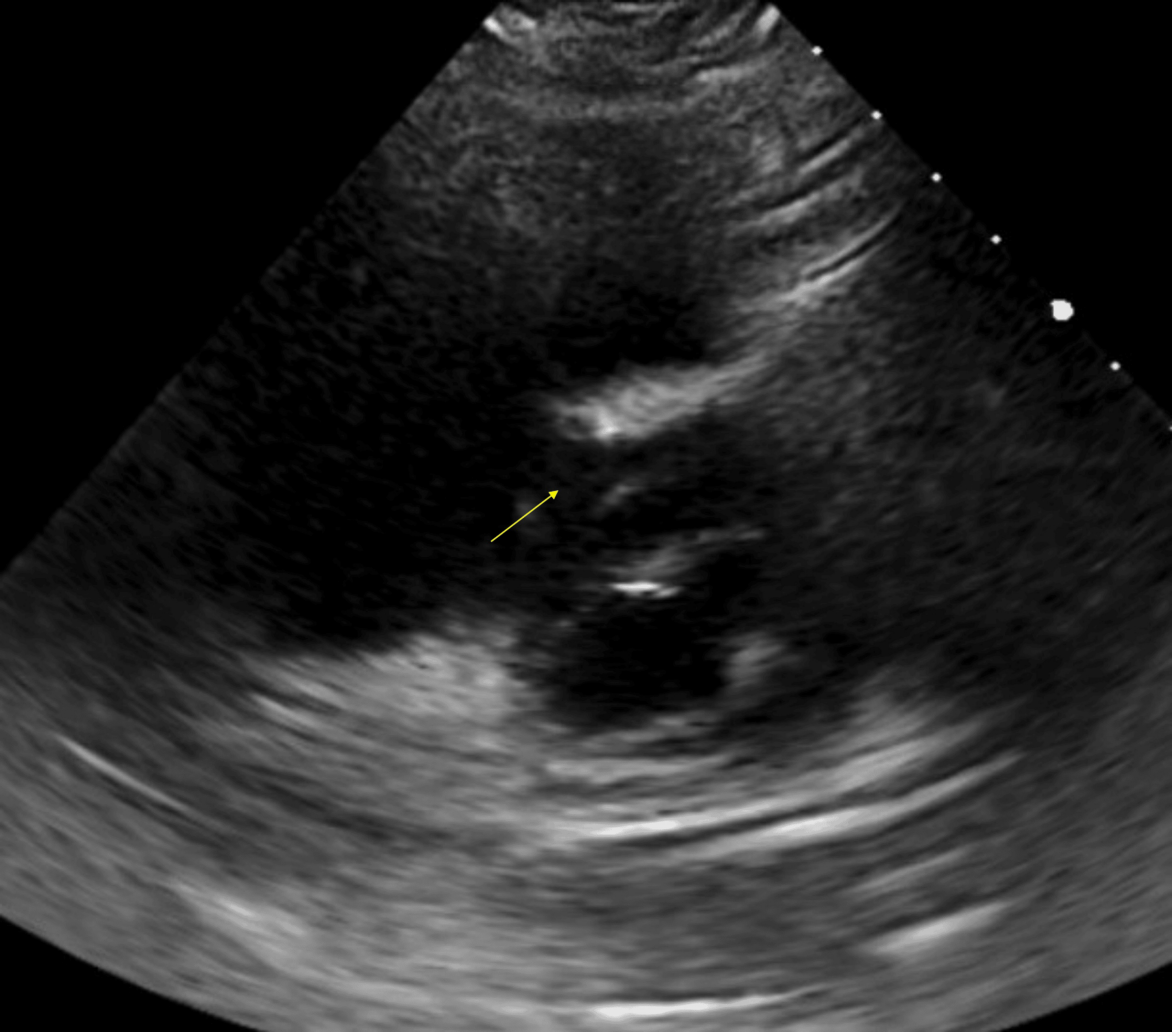
A transthoracic echocardiogram on hospital day four in parasternal short view demonstrated interventricular septal flattening (yellow arrow) during systole and right ventricle dilation consistent with acute pulmonary embolus.

**Figure 4:**
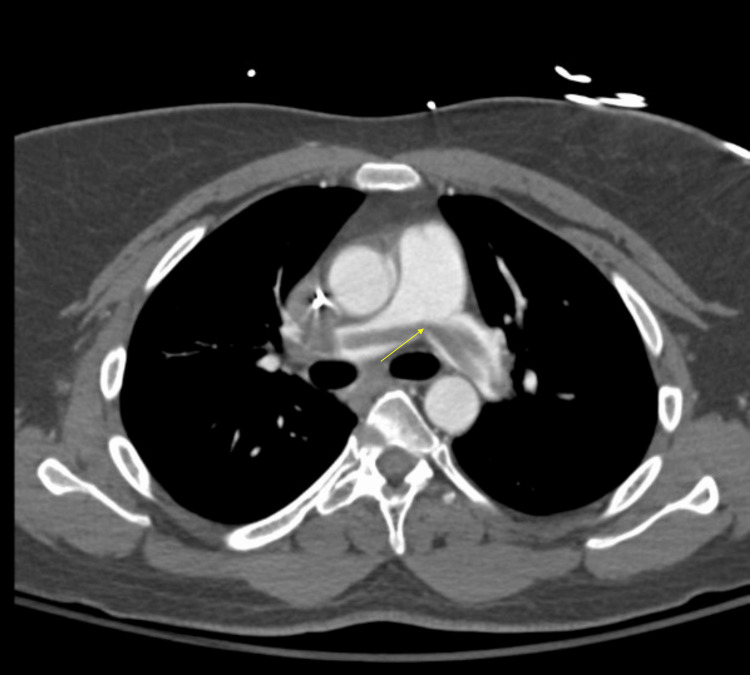
CT angiography chest on hospital day four demonstrated a large saddle pulmonary embolus (yellow arrow) extending from the main pulmonary artery to the bilateral segmental pulmonary arteries.

**Figure 5:**
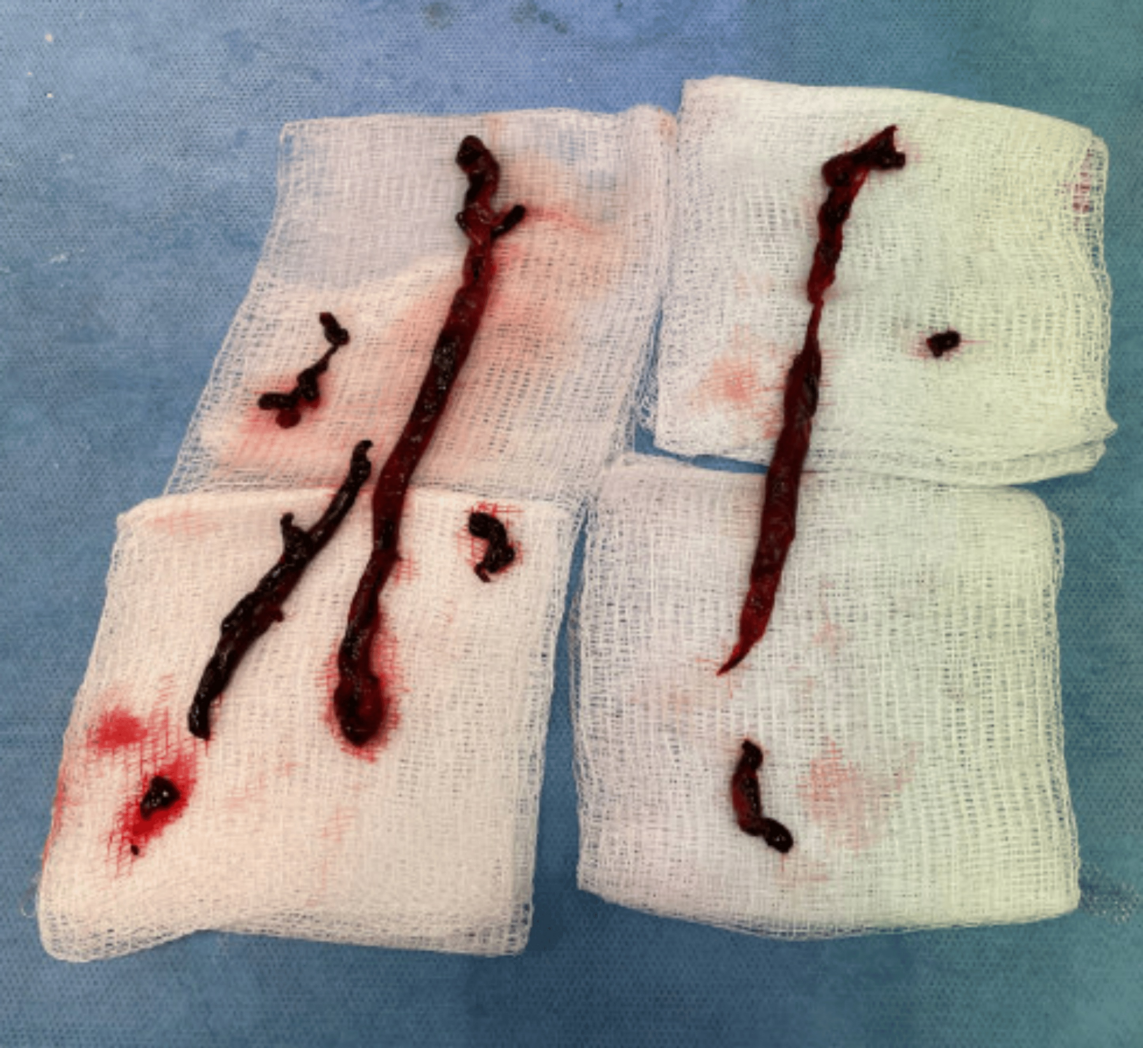
Gross image of clots retrieved during mechanical thrombectomy.

A 33-year-old woman with a history of atypical teratoid rhabdoid tumor (ATRT) status post resection and successful radiation treatment presented with one day of diplopia, blurry vision, left facial paralysis, groin numbness, and straining with urination. On magnetic resonance (MR) imaging of her central nervous system, she was found to have concerning findings of spinal and brain metastases of ATRT (Figure 1) and was admitted to the neurosurgery service for possible decompression. Given the patient’s metastases, she was deemed not a surgical candidate and plans were made for inpatient chemotherapy. According to the medication administration record, subcutaneous heparin had been administered on day zero of admission but ordered and refused for three days afterward, with nursing notes indicating that the patient had been educated on the medication’s significance. No note of the refusal was made elsewhere, including physician notes. On hospital day four, the medicine team was consulted for new-onset chest pain and tachycardia. She had an elevated high-sensitivity troponin of 53 ng/L with electrocardiogram (ECG) changes of ST elevation in aVR as well as t-wave inversions and depressions in the lateral and precordial leads, concerning for global ischemia (Figure 2). A stat transthoracic echocardiogram showed right ventricular strain (Figure 3), and follow-up computed tomography (CT) angiography of the chest revealed a large saddle pulmonary embolus extending from the main pulmonary artery to the bilateral segmental pulmonary arteries with subsequent pulmonary infarcts (Figure 4). The patient then underwent successful pulmonary artery thrombectomy (Figure 5) and inferior vena cava filter placement with successful stabilization. Notably, in speaking with the primary nurse practitioner on hospital day four, she was unaware of the patient’s refusal of heparin. On hospital day nine, the patient suddenly decompensated with noted worsening altered mental status and acute hypoxic respiratory failure. She then went into pulseless electrical activity arrest and passed away shortly afterward. Autopsy reports deemed her immediate cause of death to be respiratory failure secondary to extensive mixed organism bronchopneumonia, with the underlying cause of death to be disseminated ATRT. There was no evidence of recurrence of venous thromboembolism. 

Case 2

**Figure 6:**
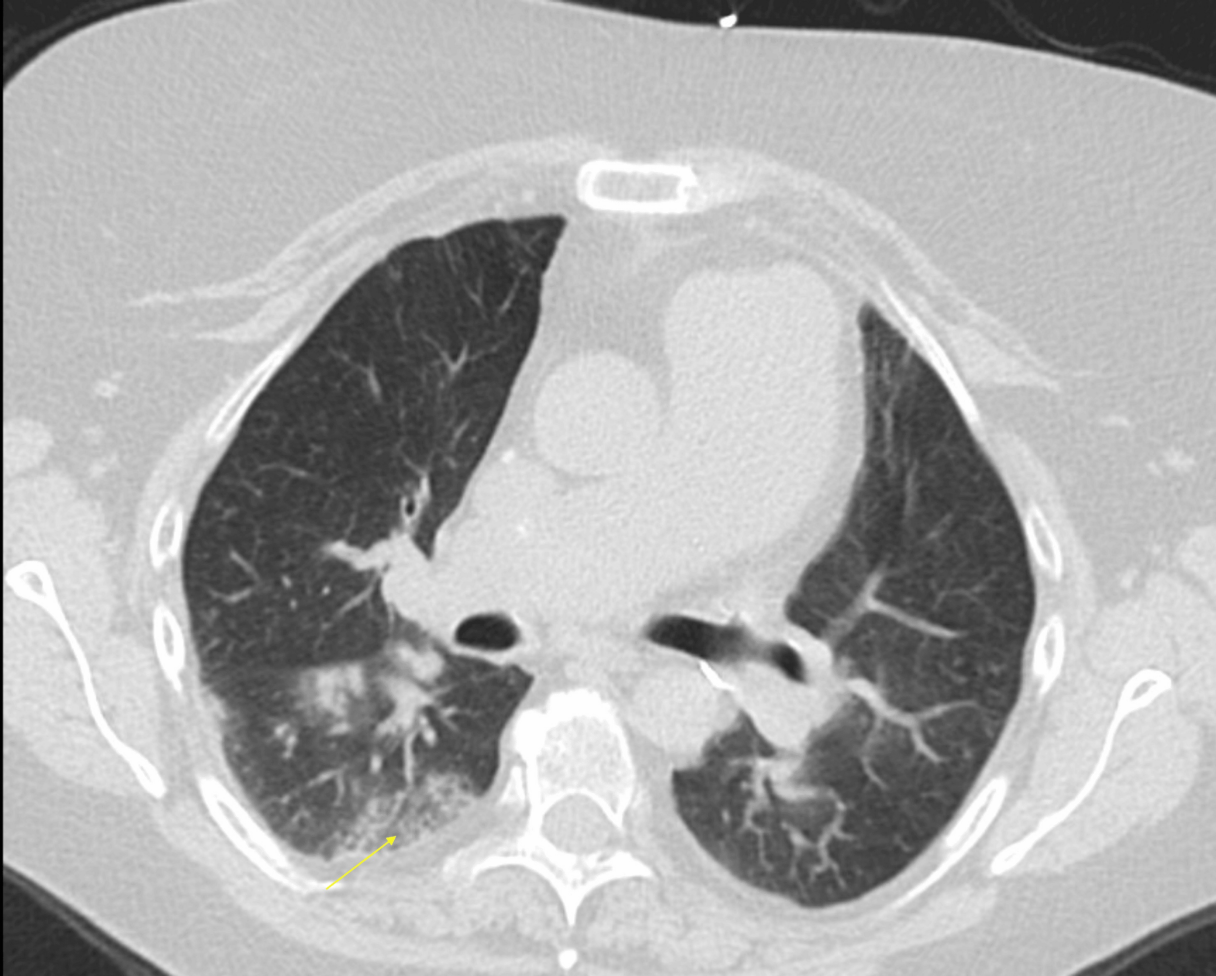
CT chest without contrast on admission demonstrating basilar air trapping consistent with possible bronchiolitis obliterans, centrilobular and subpleural glass ground-glass, and solid nodules (yellow arrow) concerning for organizing pneumonia.

**Figure 7:**
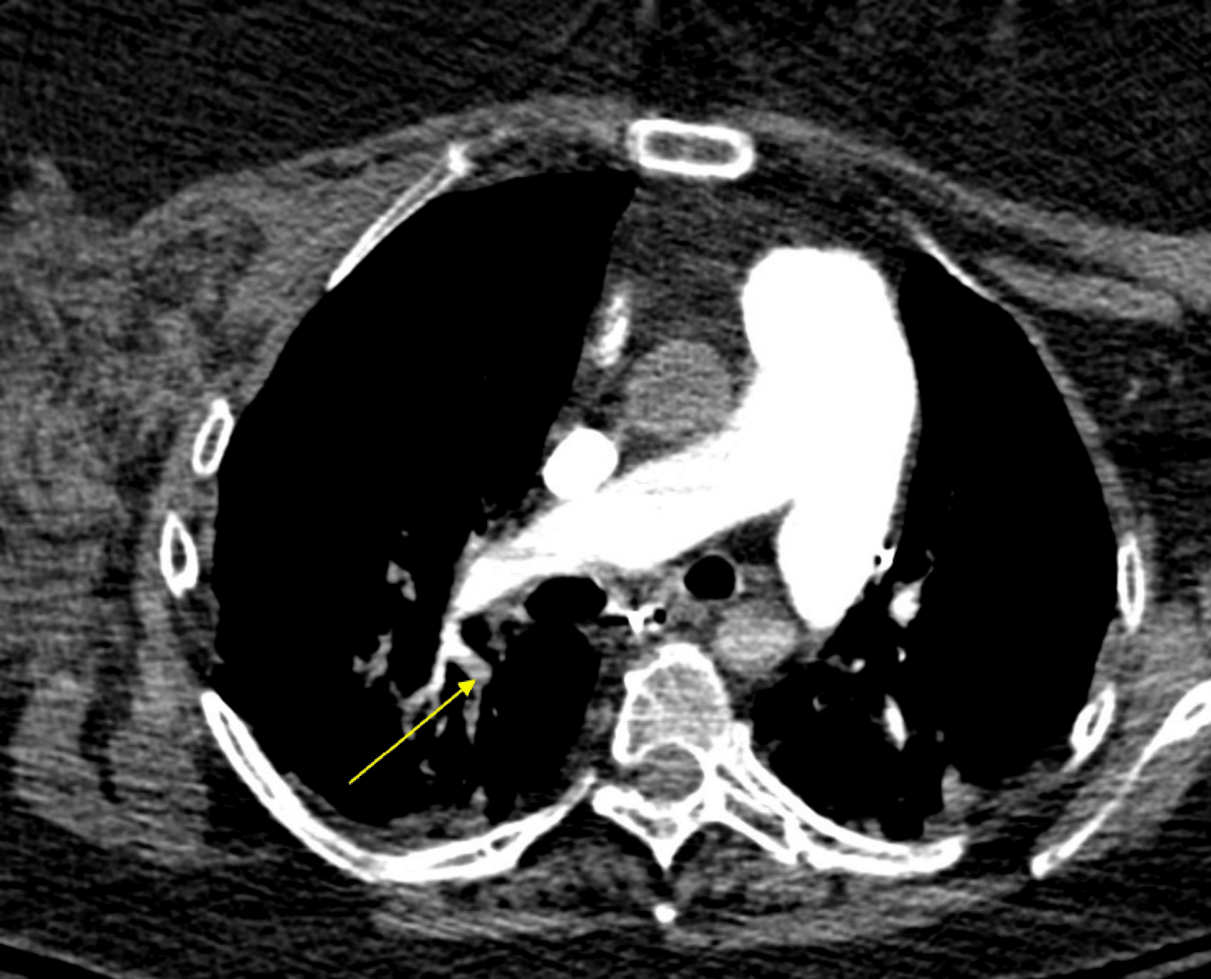
CT angiography chest on hospital day three demonstrating pulmonary emboli in the right lower lobe segmental branches (yellow arrow).

A 49-year-old woman with a history of idiopathic versus anorexigen-associated pulmonary arterial hypertension status post bilateral orthotopic lung transplantation and patent foramen ovale closure presented in acute hypoxic respiratory failure. She had a CT chest without contrast, which revealed possible bronchiolitis obliterans and subpleural ground glass nodules concerning for possible organizing pneumonia (Figure 6). At the time, a pulmonary embolus workup was not pursued. She refused subcutaneous enoxaparin for the first three days of her admission with no indication of education of the importance of medications or other acknowledgment of this refusal in the medical record. Retrospectively, when the primary team was queried about this, they stated that they were unaware that the patient had been declining enoxaparin. On hospital day three, the patient went into pulseless electrical activity arrest. Return of spontaneous circulation was achieved, and she was started on extracorporeal membrane oxygenation. A CT chest angiography revealed pulmonary emboli in the right lower lobe segmental branches, an expanding chest hematoma, and multifocal pneumonia (Figure 7). Notably, she did not have a DVT on bilateral lower extremity duplex ultrasound. She was placed on the massive transfusion protocol for suspected internal hemorrhage, then developed acidosis and probable disseminated intravascular coagulation, and ultimately was transitioned to comfort care given her poor prognosis. Her autopsy demonstrated bilateral pulmonary thromboembolism, shock liver, and kidney, no evidence of acute rejection from lung transplantation, and a focus on organizing pneumonia. The report commented that the immediate cause of death was likely bilateral pulmonary thromboembolism that led to her cardiac arrest. 

## Discussion

In both cases, VTE prophylaxis was clearly indicated due to the patients’ relative immobility and extensive medical comorbidities, which generated high-risk scores on the calculators for VTE risk integrated into our electronic medical record. In fact, the providers for both patients appropriately ordered chemical VTE prophylaxis on admission. The refusal of VTE prophylaxis should demand great attention from the care teams, given the patients’ underlying hypercoagulable conditions, secondary to malignancy in the first case and transplant-related immobility in the second. This introduces the topics of the nuanced nature of the primary team’s responsibility in following up with the lack of administration of VTE prophylaxis and the effectiveness of bedside and technological interventions to increase VTE prophylaxis adherence. In both cases, there was no documentation of VTE prophylaxis refusal in progress notes. Furthermore, in discussions with some of the primary providers, there was a lack of recognition of the absence of administration of VTE prophylaxis, although this does not confirm that all of the providers were unaware of the refusal. Nurses often face patients who refuse medications, but there should be a system in place for them to notify physicians each time a VTE prophylaxis medication is declined.

Physician and nurse perceptions of their individual responsibility for VTE prophylaxis compliance likely impact patient adherence. A study of healthcare providers’ opinions on VTE prophylaxis supports that an overwhelming majority of healthcare providers do find VTE prophylaxis important, but significantly fewer believe that appropriate VTE prophylaxis is administered all the time [2]. There is little clear delineation of the role that individual healthcare team members play in enforcing prophylaxis and properly educating patients on the risks of refusing prophylaxis. One study demonstrated that internal medicine residents were more likely to defer clinical decision-making of VTE prophylaxis to nurses [3]. Other literature has found that patients on medicine floors are also more likely to refuse VTE prophylaxis compared to other floors [4]. While these patients did have the appropriate pharmacologic prophylaxis ordered, nurses provided most of the education about VTE prophylaxis without much further intervention or acknowledgment from the collaborating physician teams. Emphasis on the gravity of missing VTE prophylaxis by both physicians and patients is substantial, as demonstrated by cases like Case 1, where a large saddle pulmonary embolus occurred after just three days of non-adherence.

While the physicians in these cases ordered medications using a VTE risk assessment calculator on admission orders, there is a dearth of appreciation of the subtleties involved in selecting which patients receive VTE administration, as most providers do not use risk assessment models such as the Caprini or Padua scores for venous thromboembolism risk stratification [2]. The Caprini score is a comprehensive risk assessment model that determines the degree and duration of pharmacologic VTE prophylaxis indicated during and after hospitalization, based on factors related to recent major events (stroke, trauma, etc.) prior to surgery and underlying venous disorders, but its use has been validated primarily in post-surgical patients [5]. The Padua prediction score is a less comprehensive risk assessment model based on factors such as age, cancer, and comorbid cardiac conditions and generates a binary score. Its use has been mainly limited to general medical inpatients [6]. While there is current debate over the general usefulness of these two frequently-used risk assessment models given the attempt to apply these scores heterogeneously to patients outside the validated population [7-9], it is clear that a stratification of risk for these patients is warranted. This lack of distinction can lead to the overuse of VTE prophylaxis for patients who may not need it, and in our cases, the underuse of VTE prophylaxis for patients who need it most. Under-appreciation of VTE importance in these patients as well as time limitations can possibly lead to team members being less diligent about following up on VTE adherence. 

Bedside interventions increasing VTE prophylaxis administration have been varied, but technology underlies many successful measures. Education of nurses and patients on the potential consequences of refusal of VTE prophylaxis has been shown to reduce missed VTE prophylaxis administrations by bridging knowledge gaps on both the provider and patient end about the necessity of VTE prophylaxis [10]. Tailoring patient education further at the bedside with an education bundle can also reduce the rates of patients refusing VTE prophylaxis [11]. This can be encompassed in a clinical decision support system (CDSS), which has also shown efficacy in increasing VTE prophylaxis administration rates [12]. Mechanical compression stockings may at times underlie a patient’s refusal to be on pharmacologic VTE prophylaxis. Mechanical prophylaxis is generally not indicated as a primary modality of prevention of VTE unless the bleeding risk is deemed too high [13]. Thus, patient and provider education should also take this point into account. This targeted patient education, especially between physician-patient, appeared to be lacking in the above cases. 

We propose a standardized protocol whereby nurses notify physicians whenever a patient refuses a high-risk medicine such as an anticoagulant. Another potential intervention inspired by these cases is the creation of a documented CDSS integrated into the electronic medical record for notifying the primary team of the lack of VTE prophylaxis administration, whether it be due to patient refusal of VTE prophylaxis or failure to order pharmacologic prophylaxis, automatically accounting for a patient’s inherent VTE risk. Once flagged, there could be a full-circle approach to the education of the patient on VTE prophylaxis between the patient and physician. As an added safety-net feature, we also propose that the physician review the medical administration record daily. By having processes for nursing-level communication to physicians, CDSS alerts, and physician ownership of the medical administration record, compliance with appropriate VTE prophylaxis would likely increase. Patient refusal after repeated education is always a possibility; however, this can be documented in a more comprehensive manner.

## Conclusions

Healthcare-associated VTE is a public health problem. These patient scenarios demonstrate that a multi-faceted quality improvement approach is necessary to increase VTE prophylaxis administration in the most vulnerable and hospitalized patients. We suggest that primary teams and physicians play a more active role in overseeing the compliance of VTE prophylaxis through adequate acknowledgment, communication, and documentation. Patients should also be strongly advised about the risks of refusing VTE prophylaxis by doctors and nurses. Management should be adjusted using evidence-based risk assessment scores for high-risk patients for primary teams and providers to follow up more aggressively on prophylaxis in order to minimize the risk of developing a VTE, which can potentially lead to a fatal outcome. 

## References

[1] Qaseem A, Chou R, Humphrey LL, Starkey M, Shekelle P (2011). Venous thromboembolism prophylaxis in hospitalized patients: a clinical practice guideline from the American College of Physicians. Ann Intern Med.

[2] Lam BD, Dodge LE, Datta S (2023). Venous thromboembolism prophylaxis for hospitalized adult patients: a survey of US health care providers on attitudes and practices. Res Pract Thromb Haemost.

[3] Piechowski KL, Elder S, Efird LE (2016). Prescriber knowledge and attitudes regarding non-administration of prescribed pharmacologic venous thromboembolism prophylaxis. J Thromb Thrombolysis.

[4] Shermock KM, Lau BD, Haut ER (2013). Patterns of non-administration of ordered doses of venous thromboembolism prophylaxis: implications for novel intervention strategies. PLoS One.

[5] Golemi I, Salazar Adum JP, Tafur A, Caprini J (2019). Venous thromboembolism prophylaxis using the Caprini score. Dis Mon.

[6] Arpaia GG, Caleffi A, Marano G (2020). Padua prediction score and IMPROVE score do predict in-hospital mortality in Internal Medicine patients. Intern Emerg Med.

[7] Hayssen H, Cires-Drouet R, Englum B (2022). Systematic review of venous thromboembolism risk categories derived from Caprini score. J Vasc Surg Venous Lymphat Disord.

[8] Hayssen H, Sahoo S, Nguyen P (2024). Ability of Caprini and Padua risk-assessment models to predict venous thromboembolism in a nationwide Veterans Affairs study. J Vasc Surg Venous Lymphat Disord.

[9] Zhou H, Hu Y, Li X, Wang L, Wang M, Xiao J, Yi Q (2018). Assessment of the risk of venous thromboembolism in medical inpatients using the Padua prediction score and Caprini risk assessment model. J Atheroscler Thromb.

[10] Haut ER, Lau BD, Hobson DB (2018). Can Nurse and Patient Education Reduce Missed Doses of Medications to Prevent Blood Clots. Patient-Centered Outcomes Research Institute (PCORI).

[11] Haut ER, Aboagye JK, Shaffer DL (2018). Effect of real-time patient-centered education bundle on administration of venous thromboembolism prevention in hospitalized patients. JAMA Netw Open.

[12] Karajizadeh M, Hassanipour S, Sharifian R, Tajbakhsh F, Saeidnia HR (2022). The effect of information technology intervention on using appropriate VTE prophylaxis in non-surgical patients: a systematic review and meta-analysis. Digit Health.

[13] Lau BD, Murphy P, Nastasi AJ (2020). Effectiveness of ambulation to prevent venous thromboembolism in patients admitted to hospital: a systematic review. CMAJ Open.

